# NCNet: Deep Learning Network Models for Predicting Function of Non-coding DNA

**DOI:** 10.3389/fgene.2019.00432

**Published:** 2019-05-29

**Authors:** Hanyu Zhang, Che-Lun Hung, Meiyuan Liu, Xiaoye Hu, Yi-Yang Lin

**Affiliations:** ^1^College of Computing and Informatics, Providence University, Taichung City, Taiwan; ^2^Labo MICS, École CentraleSup élec, Université Paris Saclay, Gif-sur-Yvette, France; ^3^Department and Graduate Institute of Computer Science and Information Engineering, Chang Gung University, Taoyuan City, Taiwan; ^4^Division of Rheumatology, Allergy and Immunology, Chang Gung Memorial Hospital, Taoyuan City, Taiwan; ^5^AI Innovation Research Center, Chang Gung University, Taoyuan City, Taiwan; ^6^Department of Computer Science and Communication Engineering, Providence University, Taichung City, Taiwan; ^7^Affiliated Cancer Hospital & Institute of Guangzhou Medical University, Guangzhou, China

**Keywords:** Non-coding DNA, residual learning, LSTM, sequence to sequence learning, deep learning

## Abstract

The human genome consists of 98.5% non-coding DNA sequences, and most of them have no known function. However, a majority of disease-associated variants lie in these regions. Therefore, it is critical to predict the function of non-coding DNA. Hence, we propose the NCNet, which integrates deep residual learning and sequence-to-sequence learning networks, to predict the transcription factor (TF) binding sites, which can then be used to predict non-coding functions. In NCNet, deep residual learning networks are used to enhance the identification rate of regulatory patterns of motifs, so that the sequence-to-sequence learning network may make the most out of the sequential dependency between the patterns. With the identity shortcut technique and deep architectures of the networks, NCNet achieves significant improvement compared to the original hybrid model in identifying regulatory markers.

## 1. Introduction

Owing to the rapid development of the next-generation sequencing (NGS) technologies, various scale sequencing data can be produced in days. Large amounts of omics data, including genomics, transcriptomics, proteomics, and metabolomics, have been accumulated rapidly. Biologists can utilize these datasets to extract knowledge (Mrozek et al., 2016; Małysiak-Mrozek et al., 2018; Mrozek, 2018). Machine learning (ML) algorithms have been applied to various bioinformatics applications (Mohri et al., 2012), resulting in significant improvement. Particularly, ML algorithms such as linear and logistic regression, random forests, hidden Markov models, Bayesian networks, Gaussian networks, and support vector machines are most commonly used in gene function prediction.

Recently, deep neural networks (DNNs), also known as deep learning, have been proved to be superior to traditional ML algorithms in most applications aimed at finding patterns from training data and building models to make predictions (Hinton et al., 2012; Krizhevsky et al., 2012). Typically supervised deep learning algorithms learn a model from a given labeled training data, then the learned model is used to predict labels for unseen data (Mohri et al., 2012). As a result of the rapid growth of hardware technologies in graphic processing units (GPU) by NVIDIA, numerous deep neural networks have been proposed. In 2012, AlexNet (Krizhevsky et al., 2012), the first deep convolutional neural network (CNN) approach using a GPU, was introduced for image classification. Since then various new architectures have been proposed including VGG (Simonyan and Zisserman, 2014), NiN (Lin et al., 2013), Inception (Chen et al., 2017), ResNet (He et al., 2015), DenseNet (Huang et al., 2016), and NASNet (Zoph et al., 2017), SENet (Hu et al., 2017). The accuracy of top-1 classification in ImageNet (Russakovsky et al., 2014) has been increased from 62.5% (AlexNet) to 82.7% (NASNet-A). All of these networks are based on a CNN. Among the recent deep learning network approaches, another useful networks are recurrent neural networks (RNNs), which have been successfully applied with tremendous success (Graves et al., 2013; Bahdanau et al., 2014; Xu et al., 2015) such as in speech recognition, neural machine translation, and image caption generation. To improve the training efficiency of RNNs, including long short-term memory (LSTM) (Hochreiter and Schmidhuber, 1997) or a gated recurrent unit (GRU) (Cho et al., 2014) has been proposed to control the gradient information in the training procedures. LSTM is one of the most well-known RNN units that has been applied in many deep learning applications (Graves et al., 2013; Kalchbrenner et al., 2015; Danihelka et al., 2016). LSTM can reduce the vanishing or exploding gradient problem in RNNs with gates that are used to memorize past information.

Since the successes are recognized by researchers, now the deep learning approaches have been introduced into bioinformatics domain to improve the performance of prediction or classification tasks. For example, CNNs surpass previous algorithms such as support vector machines or random forests in predicting the protein binding and accessibility based on a DNA sequence (Alipanahi et al., 2015). DeepSEA (Zhou and Troyanskaya, 2015) is a useful tool to predict the chromatin effects of sequence alterations with single nucleotide sensitivity. It adopts CNN to learn a regulatory sequence code from large-scale chromatin-profiling data. DeepBind (Alipanahi et al., 2015) is another useful tool to discover the sequence specificities of DNA- and RNA-binding proteins based on the patterns learned from experimental data using CNNs. In 2016, Danil et al. (Quang and Xie, 2016) proposed a DanQ model, similar to DeepSEA, but as a hybrid framework integrating a CNN and bi-directional LSTM RNN for predicting the noncoding function *de novo* from a sequence. Kelley et al. introduced Basset (Kelley et al., 2016) to serves as a tool to predict the accessibility of DNA sequences in utilizing CNNs to learn the functional activities of DNA sequences. More recently in 2017, Wei et al. proposed a DeepPSL predictor (Wei et al., 2018) based on stacked auto-encoder networks to learn high-level feature representations of proteins to predict protein subcellular localization without handcrafted features. Later in 2018, Wei et al. developed a DeepM6APred (Wei et al., 2019) predictor, which is trained on features extracted by a deep belief network together with handcrafted features by a support vector machine, to improve the ability of predicting N6-methyladenosine m6a sites. All the models above either create a new method or outperform previous existing methods in accomplishing the tasks.

In this work, we propose several enhancements of the convolutional part in the hybrid framework proposed in DanQ model. Particularly, we choose to employ the identity shortcut technique proposed in ResNet as it significantly reduces the difficulty in training a very deep neural network and successfully improves the performance of convolution networks. We confirm that the depth of deep neural networks is crucial in improving performance as deep architecture allows to build a better representation of the underlying problem than a shallow one does. We also investigate how reversing the arrangement of the convolutional and recurrent part in the hybrid framework may improve the performance.

In the remainder of this paper, we first introduce the materials that we use and describe the proposed models in section 2 in details. Then in section 3, we will report the results of evaluations of the proposed models in comparison with a reimplemented DanQ model for several metrics. In the same section, we also carefully analyze and discuss the improvements obtained by the proposed models. Costs in terms of time and space are investigated as well for the proposed models. Finally, we conclude in section 4 that the proposed models are valuable enhancements which outperform the original hybrid model, and we also suggest some possible work path in future.

## 2. Materials and Methods

### 2.1. Features and Data

Our models use the segmented GRCh37 reference genome as the data. Target TF bindings are computed from the intersections of the ChIP-seq and DNase-seq peak sets, which are uniformly processed from the ENCODE (The ENCODE Project Consortium, 2012) and Roadmap Epigenomics (Roadmap Epigenomics Consortium et al., 2015) data. Briefly, we use the same dataset that were used in the DanQ (Quang and Xie, 2016) model. The complete dataset is divided into three non-overlapping sets: training set (4,400,000 samples), validation set (8,000 samples), and testing set (455,024 samples). The former two are used in the training phase, whereas the latter one belongs to the testing phase.

**Figure 1:**
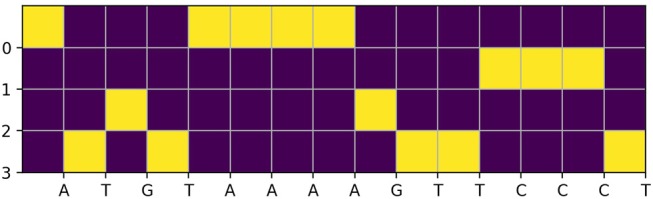
An example of one-hot encoded vector for a DNA fragment of length 15-bp.

**Figure 2:**
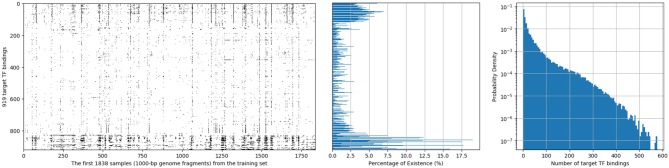
Distribution of 919 target TF bindings for the first 1,838 samples extracted from the training set of population 4,400,000 **(Left)**; Percentage of training samples that possess the corresponding TF binding for each target **(Middle)**; and a histogram of numbers of target TF bindings that training samples possess **(Right)**.

In this dataset, each 1,000-bp length input genome fragment is transformed into a 1, 000 × 4 one-hot encoded vector, part (15-bp) of an imaginary sample is shown in Figure 1. Components from top to bottom correspond to nucleobases adenine (A), cytosine (C), guanine (G), thymine (T), respectively. When one of the nucleobases appears, the corresponding component is set to one and the others are set to 0. And for each of the 1,000-bp genome fragments, 919 target TF bindings are labeled as “True” or “False” in a certain order to denote their presences or absences. An illustration of target TF bindings' distribution for the first 1,838 samples extracted from the training set is made in the left of Figure 2, in which each column represents a sample and each row represents a target TF binding, hence, a black spot denotes the existence of the horizontally relevant target for the vertically corresponding sample. We also give the probability density of every target TF binding's occurrence in the middle of Figure 2, from which we may easily deduce that the dataset is quite imbalanced, as majority of the targets are only observed in <5% of the training samples. Moreover, more than half of the targets never spread to 2.5% of the samples. And in the right of the Figure 2, we plot the histogram of the number of targets that are possessed by each sample in the training set, most of them can have only a few of the target TF bindings. The testing set has similar statistics as the training set does, therefore, we omit them here.

### 2.2. NCNet Models

We derive three novel hybrid networks, aiming at enhancing the performance of the hybrid DanQ model by employing some techniques developed recently in deep learning. We retain the main idea of introducing a bi-directional LSTM network in the framework; however, we modify the convolutional part by applying deep residual CNNs, which have been proven to be successful in many domains, such as image classification and object detections. We also attempt to reverse the arrangement of the recurrent part and convolutional part in the hybrid framework. The comparisons show that our models are either better in some aspects or comparable to the DanQ model.

#### 2.2.1. Re-implementation of DanQ Model

Our implementation uses Keras library of the latest version (2.2.4) as well as the latest version (1.0.2) of Theano backend together on Python 3.5.6 when writing this work. The original DanQ model's implementation is no longer compatible with these more recent libraries. Hence, we reimplement the DanQ model, and we shall call this model as r-DanQ model whenever we refer to it in the rest part. Then we re-build the model with the training set, and set the performance baseline with the testing set in our environments, therefore, the results are not exactly the same as in the original paper.

**Figure 3:**
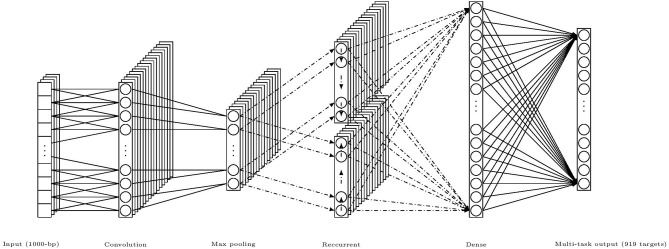
An illustration of the hybrid framework of DanQ model, (channels and nodes shown in the figure are not of exact number used in the model, but for illustrative purpose; dash-dot arrows mean that there are dropouts).

For completeness, we concisely describe the DanQ model, an illustration of the model is given in Figure 3. The Input layer regards the one-hot encoded vector of 1,000-bp genome fragments as a linear sequence of fixed length 1,000 with 4 channels and feed them to the following regular convolution layer to extract the local consecutive spatial features. And this single convolution layer constitutes the convolutional part of the hybrid framework. Then a max pooling layer is used in order to reduce the length of sequence before connecting to the bi-directional LSTM layer which constitutes the recurrent part in the framework. The main reason for including a recurrent network is that the regulatory grammar and repetitive combinations extracted by the convolutional layer maybe recognized more easily by the bi-directional LSTM because it can remember important patterns seen previously in some sense. Finally the recurrent layer is flattened and fully connected to a dense layer prepared to fully connected to the multi-task output layer of 919 nodes, each of them conducts a binary classification for the corresponding target TF bindings. There are also two dropout layers before and after the recurrent bi-directional LSTM layer respectively, which are represented by dash-dot arrows in the illustration.

#### 2.2.2. NCNet-RR Model (Residual Then Recurrent Network Model)

**Figure 4:**
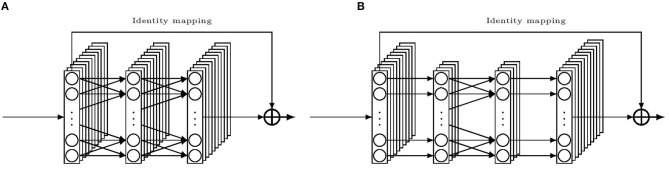
Illustration of building blocks for the residual convolution network. **(A)** a residual building block. **(B)** a “bottleneck” residual building block.

It is known that deep neural networks tend to build hierarchical features along the layers (Goodfellow et al., 2016). Base features are first constructed in the low-level convolution layers, based on which more abstract features can be built by the high-level layers, and thus, contain rich information. Furthermore, by stacking layers, the network indirectly spans the length of the kernels so that it can learn more local spatial information. However, it is more difficult to train a deep network. For a classical CNN, as shown in He et al. (2015), a shallow network yields better results than a deep network because the latter one is more prone to local optimization. However, the authors of the work found this phenomenon could be partially overcome by adding a shortcut connection linking from the beginning of a convolution block directly to the end of the same block, then combining both information before flowing to the next block. Such a block is shown in Figure 4A. Conventionally the shortcut is an identity mapping and the combination method is addition, therefore, the input and the output must be congruent. In such a case, the classical flow of a convolutional layer only needs to learn the optimal residual part of the target function, which facilitates the learning phase and helps to enable a deeper network to work much better. Such a block is called a residual block. This design made the deep residual network win the first place in the ILSVRC 2015 classification task.

Hence, we borrow the idea to enhance the convolutional part of the DanQ model with an expectation of a better result. We replace the single convolution layer with a stack of two residual blocks, in each block, the following layers are connected in order: a 1D convolution layer, then a batch normalization layer and a ReLU activation layer, finally another 1D convolution layer with another batch normalization layer. And we keep the other parts intact as in the r-DanQ model. We shall call this Residual then Recurrent network model as NCNet-RR model whenever we refer to it in the following text.

#### 2.2.3. NCNet-bRR Model (Bottleneck Residual then Recurrent Network Model)

However, a deeper network usually means more weights to train than a shallow one with similar blocks does, which leads to more time for both training and testing. This in turn limits the number of layers that can be implemented in the former model. To overcome this problem, we employ the “bottleneck” design to reduce the input/output dimensions. In Figure 4B, an illustration of the bottleneck design is given. The main idea is to use three 1D convolutions instead of two, with the first and last convolutions equipped with kernels of size one. These two convolutions simply mean to reduce and restore the channels for the middle convolution. In this way, weights are cut down in favor of more layers. We also decide to reduce the kernel size for the middle convolution compared to those in the r-DanQ model, as deep CNN may compensate the kernel size by its depth in accessing contiguous local spatial data. With such a reduction, we are able to stack 8 bottleneck residual blocks before connecting to the Recurrent part which is kept the same. Whenever refer to this bottleneck Residual then Recurrent network model later, we shall call it NCNet-bRR model.

#### 2.2.4. NCNet-RbR Model (Recurrent Then Bottleneck Residual Network Model)

In previous models, the recurrent part mostly composed by a bi-directional LSTM layer is the second part after the convolutional part, as if it is trying to learn the grammar translated by the convolutional transformations. However, it also makes sense to directly learn the grammar embedded in the raw DNA fragments by a recurrent network without a convolutional transformation. In fact, the convolutional part can then be used to interpret these globally learned patterns locally. As regions that can be accessed by a convolution node is limited by the kernel size and depth, important information may be left unknown simply because the kernel is not big enough and/or the network is not deep enough, whereas recurrent network looks into the data globally and may avoid such defaults. Hence, the rationale behind the reversion of the two network parts' arrangement is that the recurrent part may first recognize important sequences in the DNA fragments and make the convolutional part combine these global-local spatial information more easily. An analogy is that we may regard those patterns found by recurrent part as “alphabet letters,” then the convolutional part combines them into meaningful “words.” We also employs the bottleneck design of residual networks for the convolutional part, and keep other part as intact as possible. However, appropriate modification must be made to connection part, for example, the bi-directional LSTM is fed directly with the complete input sequences, no dropout is applied before the recurrent part, and a global max pooling layer is used instead of a flatten layer in connecting convolution layer to the dense layer. And we will refer to this Recurrent then bottleneck Residual network model as NCNet-RbR in the rest of the paper.

### 2.3. Training Method

All the models are initialized with the built-in Glorot uniform random initializer (Glorot and Bengio, 2010) in Keras, and then the RMSprop algorithm (Tieleman and Hinton, 2012) is applied to train the model in maximum 60 epochs with each mini-batch composing 100 samples. And the validation set is used to early stop the training phase if five consecutive epochs do not improve the loss in prevention of overfitting.

### 2.4. Environments

Our implementations are written in python 3.5.6 with Keras 2.2.4 based on Theano 1.0.2. The experiments were carried out on a computer with operating system Ubuntu 16.04.5-x86_64 with kernel version 4.4.0-131-generic, running on two Intel Xeon CPU E5-2620 v4 with 16 processors clocking at 2.10 GHz with 2 MBytes L3 cache, 256 KBytes L2 cache, 64 KBytes L1 cache, and 128 GBytes RDIMM main memory using a clock speed of 2,400 MHz. In the meanwhile, two NVIDIA Tesla P100 12GB GPUs cooperate to accelerate the experiments. The v384.130 Nvidia-driver is installed and the 7101 version of cuDNN (The NVIDIA CUDA Deep Neural Network) library lays the foundation to exploit the parallel computational power provided by the GPUs mentioned.

## 3. Results and Discussion

In this section, we present the results of evaluation of performance with the three proposed models and compare them to the performance of the r-DanQ model, which is used as the baseline. All models are tested on the same testing dataset, for each target, a separate confusion matrix is calculated, and we first compute several conventional metrics to evaluate the performance, such as accuracy, sensitivity, specificity, F1 score, and area under the Receiver operating Characteristic curve (ROC AUC) and Precision Recall curve (PR AUC). As there are 919 targets in total, it would be reasonable to use an weighted average for each of these metrics, where weights are the percentage of existence of each corresponding targets in training set after normalization, as it is meaningful to weight less on extremely imbalanced datasets than more balanced ones, see the middle part of Figure 2, notice that it is quite clear the distributions of black spots which represent positive samples are really sparse, and even the most balanced dataset's positive samples do not exceed 20%. Hence accuracy and specificity are not very useful criterion as they may retain a high value with a random predictor. Moreover, in our case, it is much more important for the presence of the target TF bindings to act as an indicator than the absence; hence, it is normal to pay more attention to other criterion. However, even with a weighted scheme, the sensitivity is much lower than the other criterion for all models, again owing to the imbalanced dataset. Therefore, we are mostly interested in how much the enhancement in convolutional part and the reverse arrangement of the framework improve the performance related to the r-DanQ mode, so we set it as baseline whose scores are all 100%.

The resulting relative performance are listed in Table 1, which generally outlines the comparison of performance among the four models. A score < 100% means the model actually performs worse than r-DanQ model for the corresponding metric. In this aspect, the NCNet-RR model is only comparable to the r-DanQ model since it only outperforms r-DanQ model in ROC AUC and PR AUC and declines a little in others, whereas the other two models generally outperform the r-DanQ model except for the specificity, at a little cost of < 1% of which, they gain a big improvement in sensitivity, which is 43.1% for NCNet-bRR model and 78.5% for NCNet-RbR model. And the F1 score is also naturally raised a lot as it essentially measures a balanced score of specificity and sensitivity. In fact, ROC AUC and PR AUC are two criterion usually considered more suitable when evaluating binary classification tasks than the former four criterion. As we use a sigmoid activation for the output layer, output may be interpreted as the probability of presence for the target, therefore they must be binarized according to a threshold to make a prediction. Thus, the ROC curve and PR curve would reflect how the other criterion would dynamically relate to the threshold when it varies from 0 to 1, so AUCs are overall measurements, whereas the other criterion only report a point performance of threshold being 0.5.

**Table 1:** Relative performance of NCNet models compared to r-DanQ model.

**Models**	**Accuracy(%)**	**Sensitivity(%)**	**Specificity(%)**	**F1 score(%)**	**ROC AUC(%)**	**PR AUC(%)**
NCNet-RR model	99.96	99.08	99.88	95.68	101.28	102.60
NCNet-bRR model	100.12	143.08	99.90	140.78	103.64	114.05
NCNet-RbR model	100.13	178.46	99.77	165.18	104.37	117.48

**Figure 5:**
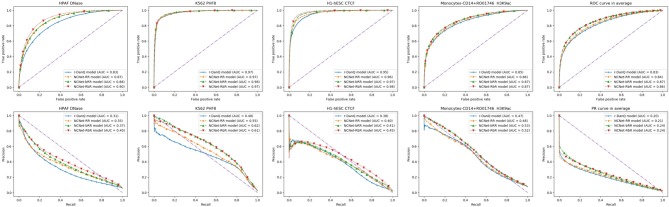
Four typical target TF binding's ROC **(Up)** and PR **(Down)** curves, with the mean ROC and PR curves (in the last column).

Since all NCNet models are preferred than r-DanQ model for ROC AUC and PR AUC, we shall look into them closely. Several individual target TF bindings' ROC and PR curves are shown row by row in Figure 5, respectively. These curves are quite typical when the target's corresponding dataset is not extremely imbalanced. We also give average results for both ROC and PR curves to the right of rows in the figure, respectively.

Generally speaking, deeper models performs better than shallow ones, as we can see in figures of ROC curves that, NCNet-bRR model and NCNet-RbR model performs roughly equal to each other, and both better than NCNet-RR model, which again is slightly better than r-DanQ model. And for PR curves, we would observe the same precedence again, but in cases, the NCNet-RbR model could even beat the NCNet-bRR model by a reasonable margin. Such a result implies that both bottleneck design and small kernels in convolution blocks in trading for depth of the network are valuable for enhancing the hybrid framework.

However, in order to consolidate this implication, only four individual observations wouldn't be enough, hence we examine all individual targets in statistical ways. We calculate the percentage of targets for which the NCNet models actually outperform the r-DanQ model on the testing dataset and report the ratio in Table 2. The result is consistent with Table 1. Notice that both of NCNet-bRR model and NCNet-RbR model perform worse than r-DanQ model on almost all targets by the angle of specificity, but as we have observed that the weighted average scores of specificity are eventually rather close to r-DanQ model, it is not difficult to see that declines in specificity for each target are negligible. In exchange, the two models are able to perform better than r-DanQ model on nearly or more than 60% of the targets in sensitivity. Such a trade is quite ideal. The ratios get even higher for the metrics of ROC AUC and PR AUC, by the two metrics, all three NCNet models manage to improve the performance on a large majority of the targets, especially for NCNet-bRR and NCNet-RbR models, the ratios achieve as high as 95%. Therefore, the observations firmly support the implication mentioned above.

**Table 2:** Percentage of targets that perform better with NCNet models than r-DanQ model.

**Models**	**Accuracy(%)**	**Sensitivity(%)**	**Specificity(%)**	**F1 score(%)**	**ROC AUC(%)**	**PR AUC(%)**
NCNet-RR model	20.67	13.71	31.12	14.80	76.71	67.46
NCNet-bRR model	54.52	64.74	3.92	65.29	98.59	95.42
NCNet-RbR model	52.56	58.54	2.94	58.76	92.49	94.89

**Figure 6:**
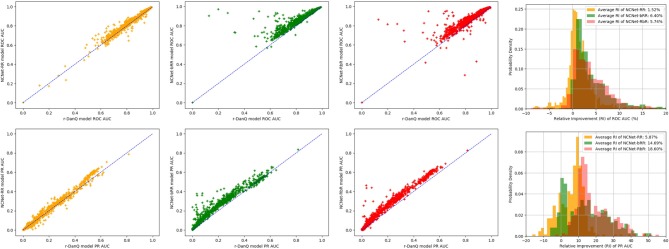
Scatter comparison to r-DanQ model for ROC AUC **(Top)** and PR AUC **(Bottom)** of NCNet-RR model (First), NCNet-bRR model (Second), NCNet-RbR model (Third) and an overlapped histogram of relative improvements for the three NCNet models (Forth).

An interesting point is that NCNet-bRR model beat r-DanQ model on more targets than the NCNet-RbR model does on all metrics, yet NCNet-RbR model eventually has higher weighted average scores than NCNet-bRR model on most metrics. Therefore, visualizations of the extent of improvements are realized by a series of scatter comparisons between NCNet models and r-DanQ model for ROC AUC and PR AUC, so that we may also investigate exactly how much improvement are made for each target by NCNet models. See Figure 6, points above the anti-diagonal segment represents the targets on which the NCNet models outperform r-DanQ model, further the point is vertically away from the anti-diagonal segment, bigger the improvement is. In the figure two overlapped histograms of relative improvements for ROC AUC and PR AUC, respectively, are also given. We should add a remark for those points near the left-bottom corner for PR AUC as they stand for small values of AUC, and we exclude those points if they are less than 0.2 for r-DanQ model in calculating the histogram. Though NCNet-bRR model possess more targets on which the performance is better than r-DanQ model, it is clear now that, NCNet-RbR generally improves more than NCNet-bRR model on those targets that defeat r-DanQ model, especially for PR AUC, which explains our interesting observation. And we conjecture that passing data through max pooling layer and dropout layer before to the bi-directional LSTM layer, some useful information may be lost, and which could have been captured if raw data are directly fed to the recurrent layer.

In addition to the comparisons of convolutional metrics above, we also consider the cost in terms of both the time and space, as listed in Table 3. The modification to the convolutional part of NCNet-RR model is disappointed as it takes much more time to train or to make prediction, and it requires more space to store the model than the r-DanQ model, but only brings limited improvements, which suggests that the depth of convolution network is crucial to the performance. And for NCNet-RbR model, the modification leads to even more time than that NCNet-RR model needs, due to the width of recurrent layer as we feed it with raw DNA fragments. However, the improvements can not be ignored and the model size is reduced to <5% of r-DanQ model. Therefore, it is suitable for cases where storage is the main concern, and it remains to be potential with developments in fast training recurrent networks. On the contrary, the enhancement introduced in NCNet-bRR model is quite successful in both cost of time and space. Compared to r-DanQ model, it not only takes <20% space to store the model, but also cuts down half of the time to train the model or two thirds of the time to predict for unseen samples. And take the similar performance as NCNet-RbR model in consideration, it should be the best option in most cases.

**Table 3:** Time and Spatial cost of the models.

**Models**	**Model size**	**Training time per epoch(h)**	**Testing time(s)**
r-DanQ model	375.4 MB	~ 4	~ 1,704
NCNet-RR model	465.5 MB	~ 19	~ 2,648
NCNet-bRR model	69.5 MB	~ 2	~ 617
NCNet-RbR model	18.1 M	~ 42	~ 6,790

## 4. Conclusion and Perspectives

In this work, we mainly explore the modification of the convolutional part of the hybrid framework with a deep residual network to enhance the performance. In conclusion, all the proposed NCNet models outperform the r-DanQ model for identifying the TF binding sites directly from noncoding DNA fragments for metrics of ROC AUC and PR AUC, especially the NCNet-bRR model and NCNet-RbR model. Therefore their outputs are appropriate candidates as input data for the following phases in DeepSEA to predict the chromatin effect or variant functionality. We also confirmed that depth of the convolution network is crucial for improving performance. Since the bottleneck design and small kernels of the network are effective techniques in trading for depth, they should be considered whenever possible. Moreover, we make the most out of the residual convolution network when it runs deep. Besides, when the employment of bottleneck design and small kernels may even gain us time and space if the whole hybrid framework is connected appropriately. We also declare that it is possible to reverse the arrangement of the convolutional and recurrent part of the hybrid framework to maintain similar performance or even achieve better results, though the reversed model may take much longer time to train or to predict when effectively reducing the model size.

With the success of applying the idea of residual convolution network in the hybrid framework, we believe that other powerful convolution networks could play the role as well as the residual network or even better in principle. And this kind of thoughts may also applied to the recurrent part by some enhancement too. Thus, we suggest to test other combinations of convolution network and recurrent network to make performance improvements in future. Moreover, as the arrangement of different part of the framework matters, one may even add multiple convolutional and recurrent part to the hybrid framework and try different arrangements of them.

## Data Availability

The datasets generated for this study can be found in ENCODE (The 83 ENCODE Project Consortium, 2012), https://www.encodeproject.org/ or DanQ model (Quang and Xie, 2016); p.213 http://github.com/uci-cbcl/DanQ.

## Author Contributions

HZ and C-LH designed the models, experiments, and revised the manuscript. HZ implemented the models. C-LH wrote the Introduction section, whereas HZ wrote the remaining part of manuscript text. Y-YL carried out these experiments. ML and XH verified the experimental results.

### Conflict of Interest Statement

The authors declare that the research was conducted in the absence of any commercial or financial relationships that could be construed as a potential conflict of interest.
