# Corrigendum: NCNet: Deep Learning Network Models for Predicting Function of Non-Coding DNA

**DOI:** 10.3389/fgene.2019.00923

**Published:** 2019-09-13

**Authors:** Hanyu Zhang, Che-Lun Hung, Meiyuan Liu, Xiaoye Hu, Yi-Yang Lin

**Affiliations:** ^1^College of Computing and Informatics, Providence University, Taichung City, Taiwan; ^2^Labo MICS, École CentraleSup élec, Université Paris Saclay, Gif-sur-Yvette, France; ^3^Department and Graduate Institute of Computer Science and Information Engineering, Chang Gung University, Taoyuan City, Taiwan; ^4^Division of Rheumatology, Allergy and Immunology, Chang Gung Memorial Hospital, Taoyuan City, Taiwan; ^5^AI Innovation Research Center, Chang Gung University, Taoyuan City, Taiwan; ^6^Department of Computer Science and Communication Engineering, Providence University, Taichung City, Taiwan; ^7^Affiliated Cancer Hospital & Institute of Guangzhou Medical University, Guangzhou, China

**Keywords:** Non-coding DNA, residual learning, LSTM, sequence to sequence learning, deep learning

In the published article, there was an error in affiliation “1.” The affiliation “Affiliated Cancer Hospital & Institute of Guangzhou Medical University, Guangzhou, China” should be moved to affiliation “7” and should be removed for the first and corresponding authors. Additionally, all subsequent affiliations should move up in order.

The authors apologize for this error and state that this does not change the scientific conclusions of the article in any way. The original article has been updated.

